# Testing of therapies in a novel nebulin nemaline myopathy model demonstrate a lack of efficacy

**DOI:** 10.1186/s40478-018-0546-9

**Published:** 2018-05-30

**Authors:** Tamar E. Sztal, Emily A. McKaige, Caitlin Williams, Viola Oorschot, Georg Ramm, Robert J. Bryson-Richardson

**Affiliations:** 1 School of Biological Sciences, Monash University, Melbourne, Australia; 2 Monash Ramaciotti Centre for Cryo Electron Microscopy, Monash University, Melbourne, VIC 3800 Australia; 3 Department of Biochemistry and Molecular Biology, Monash University, Melbourne, Australia; 4 Biomedicine Discovery Institute, Monash University, Melbourne, Australia

**Keywords:** Nebulin, Nemaline myopathy, Zebrafish, Treatment

## Abstract

Nemaline myopathies are heterogeneous congenital muscle disorders causing skeletal muscle weakness and, in some cases, death soon after birth. Mutations in *nebulin*, encoding a large sarcomeric protein required for thin filament function, are responsible for approximately 50% of nemaline myopathy cases. Despite the severity of the disease there is no effective treatment for nemaline myopathy with limited research to develop potential therapies. Several supplements, including L-tyrosine, have been suggested to be beneficial and consequently self-administered by nemaline myopathy patients without any knowledge of their efficacy. We have characterized a zebrafish model for nemaline myopathy caused by a mutation in *nebulin*. These fish form electron-dense nemaline bodies and display reduced muscle function akin to the phenotypes observed in nemaline myopathy patients. We have utilized our zebrafish model to test and evaluate four treatments currently self-administered by nemaline myopathy patients to determine their ability to increase skeletal muscle function. Analysis of muscle pathology and locomotion following treatment with L-tyrosine, L-carnitine, taurine, or creatine revealed no significant improvement in skeletal muscle function emphasizing the urgency to develop effective therapies for nemaline myopathy.

**Electronic supplementary material:**

The online version of this article (10.1186/s40478-018-0546-9) contains supplementary material, which is available to authorized users.

## Introduction

Nemaline myopathies are congenital muscle diseases characterized by the presence of nemaline (rod-like) bodies that form within the skeletal muscles. The disease presents with clinically heterogeneous phenotypes, ranging from adult onset mild muscle weakness to, in severe cases, death in utero or just after birth [43]. Causative mutations have now been identified in 11 different genes, encoding components that form or regulate the thin filament (*ACTA1* [27], *NEB* [31], α-tropomyosin [18], β-tropomyosin [8], troponin T1 [14], cofilin [1], *KBTBD13* [34], *KLHL40* [32], *KLHL41* [11], *leiomodin-3* [45], and *MYPN* [24]).

*Nebulin* (*NEB*) plays an important role in regulating thin filament length and is the most frequently affected gene in nemaline myopathy, accounting for approximately 50% of all cases [2]. Mutations in *NEB* result in nemaline bodies throughout the muscle combined with diminished contractile strength and force generation [21, 31]. Deletion of exon 55, causing a common form of autosomal recessive nemaline myopathy [19], results in shortened thin filaments, alterations in crossbridge cycling kinetics, and reduced calcium-sensitivity following loss of NEB [28, 30]. Similarly, a reduction in Nebulin in zebrafish recapitulates many of the clinical and pathological aspects of nemaline myopathy observed in patients [38, 39].

The lack of an effective treatment for nemaline myopathy has resulted in many patients and their families testing compounds on an ad-hoc basis, with a number of compounds listed on patient support websites (http://www.nemaline.org/resources/drugs.html). Perhaps the most promising of these is L-tyrosine, a non-essential amino acid, either derived from the diet or synthesised in the liver from phenylalanine, which functions as a precursor to several neurotransmitters and hormones including adrenaline and dopamine [5]. Kalita (1989) reported improvements in muscle strength and appetite and reductions in pharyngeal secretions after he and his son received daily supplementations of L-tyrosine. Within 10 days of L-tyrosine withdrawal there was a decrease in muscle strength suggesting that the improvement observed resulted from L-tyrosine treatment and was not sustained [15]. In a small-scale trial, five patients received L-tyrosine doses from 250 to 3000 mg/day for a period of 2 months to 5 years [33]. Following L-tyrosine treatment, all patients reported varying short-term improvements in muscle strength and ‘energy’ levels, however due to various limitations (no placebo group, large age variability, and variable disease mutations), no firm conclusions could be made as to efficacy [33]. L-tyrosine doses were also administered to a mouse model of nemaline myopathy (*ACTA1*^H40Y^) for 4 weeks, and although treatment was reported to partially alleviate mobility deficits and decrease nemaline bodies [26], the long-term benefits of L-tyrosine supplementation were not determined.

We have established a zebrafish model of NEB nemaline myopathy to test the ability of existing supplements, currently self-administered by patients, to improve skeletal muscle function. We show that zebrafish *neb*^*−/−*^ mutants exhibit a reduction in birefringence, resulting from disruption of muscle structure, the formation of electron dense nemaline bodies, as well as Actinin2 and F-actin positive aggregates throughout their muscle fibres, analogous to patient biopsies. The NEB nemaline myopathy model display decreased muscle function which cannot be improved by treatment by L-tyrosine, taurine, L-carnitine, or creatine. This suggests that existing treatments are ineffective in improving skeletal muscle performance in NEB nemaline myopathy, highlights the need for further research into novel therapies, and provides a model to assist in their identification.

## Methods

### Fish strains and maintenance

Zebrafish were maintained according to standard protocols [44]. Zebrafish strains used were Tg(*neb*^*+/−*^*; Lifeact-eGFP*) [4] and an ENU-generated *neb* mutant line (sa906), obtained from the Zebrafish International Resource Centre. Allele specific PCR KASP technology (Geneworks) was used for *neb* genotyping.

### cDNA synthesis and RT-PCR

Total RNA was extracted using TRIzol reagent (Invitrogen Life Technologies). RNA samples were treated with RQ1 RNase-free DNase (Promega). cDNA was synthesized from 1 μg of each RNA sample in a 20 μl reaction using Protoscript first strand cDNA synthesis kit (New England Biosciences) and oligo(dT)20 primer following the supplier’s instructions. Primers used for RT-PCR were (*neb*F: TGAGCACAACTACCGCACTC, *neb*R: GAACCTTTGAGGCCA TTTTG, *βAct*F: GCATTGCTGACCGTATGCAG, *βAct*R: GA TCCACA TCTGCTGGAAGGTGG).

### Histology and antibody staining

For Gomori trichrome staining, 6 dpf zebrafish were anesthetized and heads were cut for genotyping. Tails were snap frozen and sections (10 μm) were cut using a Leica CM 1850 cryostat. Sections were then stained with modified Gomori trichrome and imaged using a 63× 1.4 numerical aperture oil immersion objective. Fiber area was measured using Fiji [35]. Antibody staining was performed as described in [38]. Antibodies used were anti-α-Actinin2 (Sigma clone A7811, 1:100), rhodamine tagged phalloidin (Molecular Probes, 1:200), and AlexaFlour-488-labelled secondary antibody (Molecular Probes, 1:200).

### Electron microscopy

Zebrafish were fixed according standard procedures in 2.5% glutaraldehyde, 2% paraformaldehyde in 0.1 M sodium cacodylate buffer. Post-fixed with 1% OsO_4_, 1.5% K_3_Fe(III)(CN)_6_. Samples were dehydrated in ethanol and the zebrafish were flat embedded in Epon 812. Ultrathin sections of 70 nm were cut on a Leica Ultracut UCT7 and stained with uranyl acetate and lead citrate. Large area EM tile sets were taken on a FEI NovaNanoSEM 450 equipped with an ETD secondary electrons in-lens detector set at 10 kV and a STEM II (HAADF) detector set at 30 kV. MAPS 2.1 software was used to create the tile sets. High resolution EM imaging was done on a Hitachi 7500 TEM and a FEI Tecnai 12 TEM.

### Muscle function assays

Touch evoke and locomotion assays were performed on 2 and 6 dpf zebrafish respectively as per [37]. For dosage analyses on wildtype zebrafish, an inactivity threshold of 6 mm/s, detection threshold of 25 mm/s and maximum burst threshold of 30 mm/s were used. For the NEB nemaline myopathy zebrafish model, an inactivity threshold of 1 mm/s, detection threshold of 30 mm/s and maximum burst threshold of 30 mm/s were used. The total distance swum above the inactivity threshold and below maximum burst threshold in a 10-min period were extracted using the ZebraLab software (ViewPoint Life Sciences). Blinding of treatments groups was used in combination with randomization of both the position of the fish within the plates and screening order of plates to remove any bias. Once the testing and genotyping was completed the treatments groups were uncovered.

### Toxicology analyses

For dosage analyses, L-tyrosine disodium salt hydrate (T1145, Sigma), taurine (T0625, Sigma), L-carnitine inner salt (C0158, Sigma), and creatine monohydrate (C3630, Sigma) were all dissolved in water at a concentration of 250 mM and diluted appropriately to ensure that 1 ml of each chemical was added to 24 ml of embryo medium in a 90 cm petri dish. Thirty wildtype Tübingen embryos aged 28 h post fertilization (hpf) were dechorionated and placed in the petri dish. For control treatments, 1 ml of water alone was added to 24 ml of embryo medium. Zebrafish were treated from 28 hpf until 6 dpf. Treatments were changed daily and zebrafish were monitored for survival, heart rate, and swimming performance as indicators of toxicity. Four independent treatments were performed for tyrosine and three independent treatments were performed for taurine, L-carnitine, and creatine. The resting heart rates were measured at 2 dpf by counting the number of heart beats in 10 s. Heart rate measurements were performed in triplicate with 10 fish per experiment. For heart rate and swimming assays all treatments were blinded and randomized to avoid experimental bias. Once the testing and analyses were completed the treatments groups were revealed.

### Chemical treatments

For treatment of the NEB nemaline myopathy zebrafish model, Tg(*neb*^*+/−*^*; Lifeact-eGFP*) or *neb*^*+/−*^ adult fish were incrossed and the resultant progeny were dechorinated at 24 hpf and 30 embryos were placed in either chemically or water treated embryo at 28 hpf. Treatments were changed daily from 28 hpf water until 6 dpf at which time wildtype embryos were transferred to 24-well plates and NEB nemaline myopathy zebrafish were transferred into 48-well plates for locomotion assays. For distance assays, based on the SD (0.178±0.146) of the untreated *neb*^*−/−*^ mutant and the smallest n for the drug treated mutant fish groups (206) fish this gave us 0.80 power at 0.05 significance to detect an improvement of 20%. For speed assays, based on the SD (0.496±0.176) of the untreated *neb*^*−/−*^ mutant fish this gave us 0.80 power at 0.05 significance to detect an improvement of 9%.

### Statistics

**Fig. 1:**
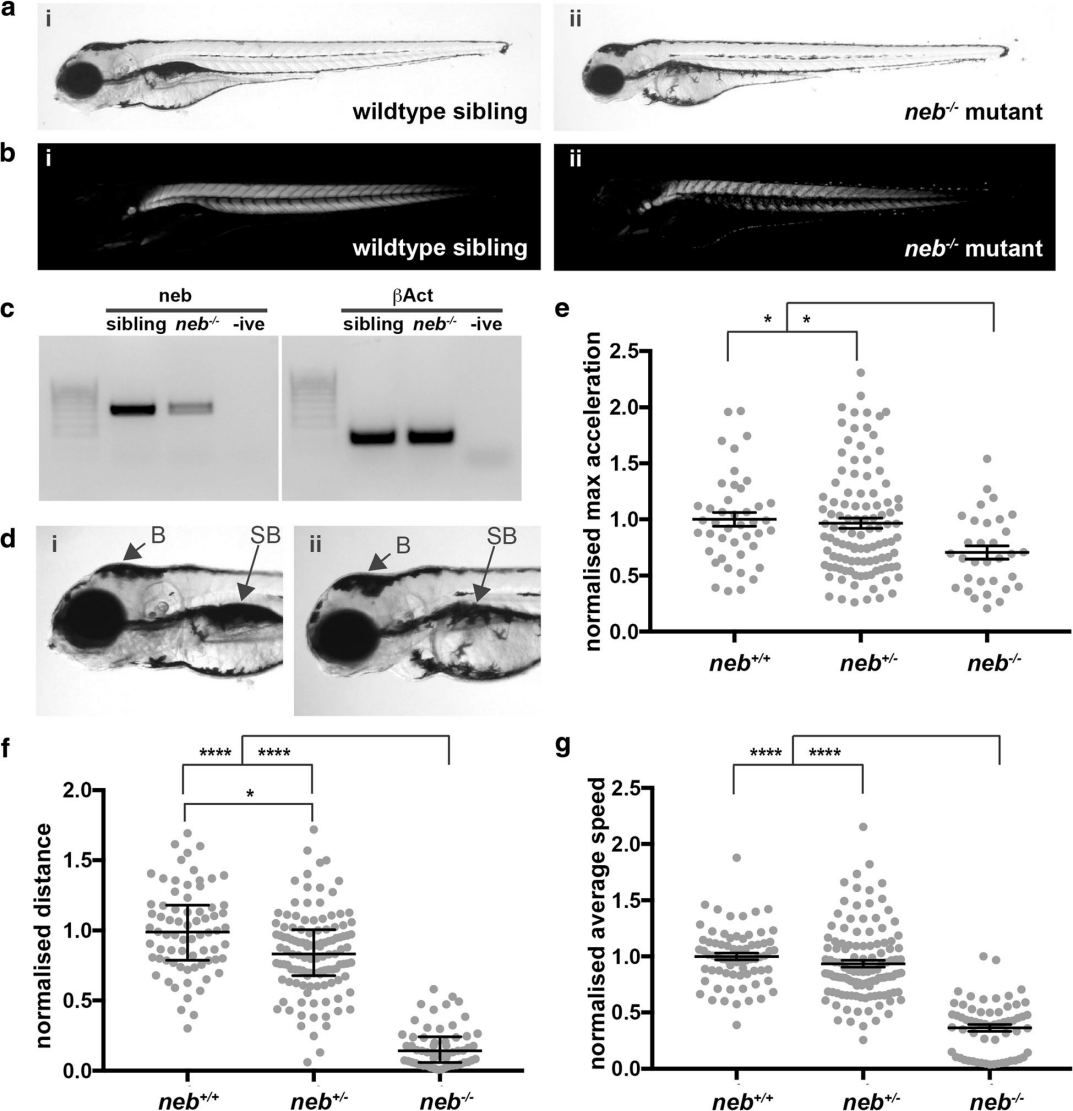
Characterization of the *neb* (sa906) mutant zebrafish strain. a & b) At 4 dpf *neb*^−/−^ zebrafish (aii) appear smaller in size and (bii) display a loss of birefringence compared to their wildtype siblings (ai & bi). c) RT-PCR analysis in and *neb*^−/−^ mutant embryos at 2 dpf shows a reduction in *neb* mRNA levels compared to wildtype siblings (sibling). *βAct* was used as a positive control. d) ii) *neb*^−/−^ mutants display a smaller eye, brain region (B) and deflated swim bladder (SB) compared to their i) wildtype siblings. e) Quantification of the maximum acceleration recorded from touch-evoked response assays of *neb*^−/−^ fish compared to wildtype siblings at 2 dpf. Error bars represent mean±SEM for three independent experiments (*n* = 11,9,12 *neb*^*−/−*^, 55,25,32 *neb*^*+/−*^, 18,13,12 *neb*^*+/+*^ zebrafish per experiment), **p < 0.01. f & g) Quantification of the normalized (f) distance and (g) speed travelled by *neb*^−/−^ mutants compared to wildtype siblings at 6 dpf. For f) error bars represent median±interquartile range for three independent experiments (for *n* = 19,23,19 *neb*^*−/−*^, 41,42,36 *neb*^*+/−*^, 31,20,21 *neb*^*+/+*^ zebrafish). For g) error bars represent mean±SEM range (for n = 19,23,14 *neb*^*−/−*^, 41,42,36 *neb*^*+/−*^, 30,20,21 *neb*^*+/+*^ zebrafish per experiment). *p < 0.5, **** *p* < 0.001

**Fig. 2:**
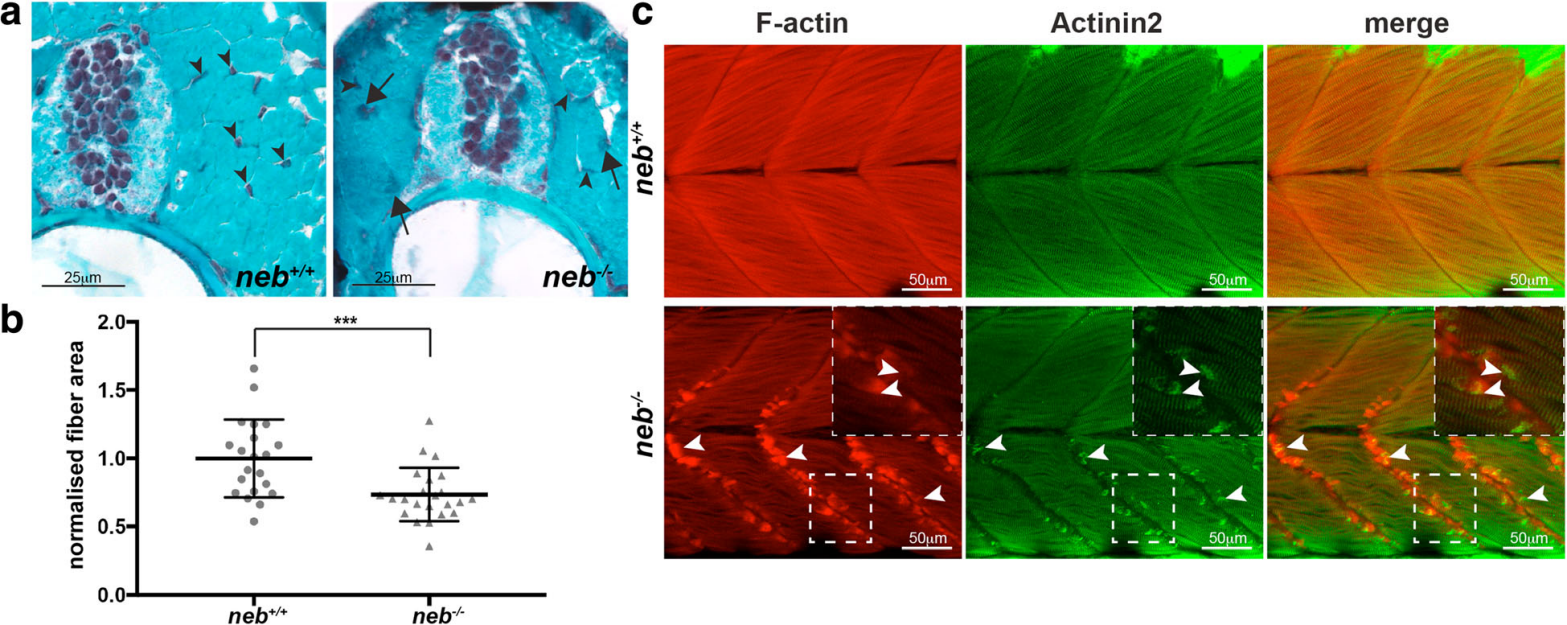
Characterisation of skeletal muscle pathology in *neb*^−/−^ fish. **a** Gomori trichome staining of *neb*^−/−^ skeletal muscle sections reveal the presence of dark regions (arrows) throughout the muscle indicative of nemaline bodies not observed in *neb*^*+/+*^ fish. Nuclei (arrowhead) are evenly organized in *neb*^*+/+*^, however, appear disorganized in *neb*^−/−^ fish. **b** Quantification of normalized fiber area from Gomori trichome stained sections in *neb*^−/−^ (*n* = 23 fibers) compared to *neb*^+/+^ fish (*n* = 21 fibers). Error bars represent mean±SD, *** *p* < 0.001. **c** *neb*^−/−^ mutants exhibit F-actin (red) and Actinin2 (green) positive aggregates at the myosepta (arrowheads) (and zoomed inset) compared to wildtype siblings at 2 dpf

For toxicology analyses on wildtype fish, all values were normalized to water supplemented wildtype fish in the same replicate. Normality of data was determined using a D’Agostino and Pearson test for normality. For toxicology analyses on wildtype fish, normal data (Additional file 1: Figures S2, S3, and S4a and b) was analysed by one-way ANOVA using Dunnett’s correction for multiple comparisons. For data failing the normality test (Additional file 1: Figure S4C), the test was repeated after the data was logtransformed which did not result in a normal distribution of data. Therefore, data from the three replicates was pooled and a Kruskal-Wallis test was performed and correction for multiple comparisons conducted using Dunn’s test. For swimming analyses and fiber area quantification on the NEB nemaline myopathy model, all values were normalized to the average water supplemented *neb*^*+/+*^ siblings. Normality of data was determined using a D’Agostino and Pearson test for normality and normal data (Fig. 1e, g and 2b) was analysed using an unpaired t-test or one-way ANOVA using Dunnett’s correction for multiple comparisons. For data failing the normality test (Fig. 1f, Fig. 4, Additional file 1: Figures S5 and S6), the test was repeated after the outliers were removed by the ROUT method (Q = 1%) or the data was logtransformed. In neither case did this result in a normal distribution of data. Therefore, in these cases the data from the three replicates was pooled and a Kruskal-Wallis test was performed and correction for multiple comparisons conducted using Dunn’s test. For phenotypic analyses (Fig. 5b, c and Additional file 1: Figure S7), the results of the three replicates were used to determine the mean percentage of each phenotype and to plot the graphs. The proportion of the phenotypes was determined by pooling the data from all three replicates and conducting a Chi-square test for each treatment against its respective control. All statistical analyses were conducted using GraphPad Prism 7.

## Results

### *neb* mutants display a reduction in muscle function

We obtained a zebrafish mutant strain (sa906) which contains a point mutation in *neb* causing a nonsense mutation in exon 30 (of 134) of the transcript. We have verified the mutation in this strain and show, using RT-PCR, that this leads to a reduction in *neb* mRNA in *neb*^*−/−*^*fish* compared to their wildtype siblings (Fig. 1c). Morphologically, *neb*^*−/−*^ mutants are smaller than their wildtype siblings and display a thinner trunk region (Fig. 1a). *neb*^*−/−*^ mutants also show a loss of birefringence at 4 days post fertilization (dpf) compared to wildtype siblings indicating that their sarcomeric muscle structure is disrupted (Fig. 1b). To determine whether loss of Neb results in impaired skeletal muscle function, we performed touch evoke and locomotion analyses to measure swimming performance. *neb*^*−/−*^ mutants display a significant reduction in maximum acceleration (proportional to muscle force) at 2 dpf (*neb*^*−/−*^ = 0.66±0.32 SEM, *neb*^*+/−*^ = 0.92±0.49 SEM, *neb*^*+/+*^ = 1.0±0.49 SEM; Fig. 1e), in distance travelled at 6 dpf (*neb*^*−/−*^ = 0.16±0.08 SEM, *neb*^*+/−*^ = 0.84±0.17 SEM, *neb*^*+/+*^ = 1.0±0.17 SEM; Fig. 1f) and speed at 6 dpf (*neb*^*−/−*^ = 0.49±0.10 SEM, *neb*^*+/−*^ = 0.94±0.19 SEM, *neb*^*+/+*^ = 1.0±0.14 SEM; Fig. 1g) compared to wildtype siblings. Interestingly, we observe that *neb*^*−/−*^ mutants fail to inflate their swim bladder by 4 dpf (Fig. 1a&d), another indicator of impaired muscle function.

### *neb*^*−/−*^ mutants display nemaline bodies and actin accumulation

**Fig. 3:**
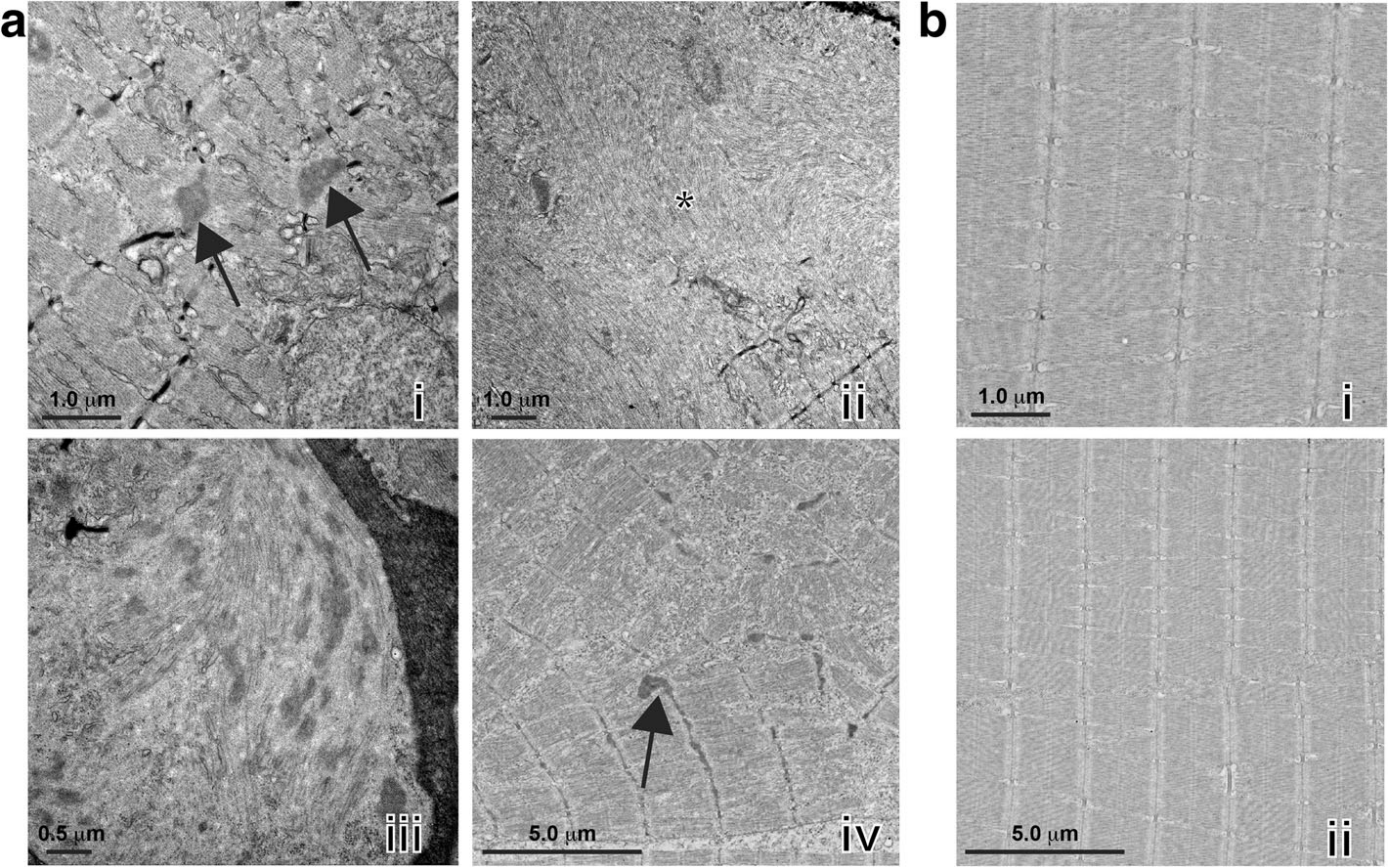
Examination of *neb*^−/−^ skeletal muscle by electron microscopy. a) *neb*^*−/−*^ mutant skeletal muscles display (i, iv) thickened Z-disks (arrows), (ii) fiber breakage (asterisks), (iii) accumulations of nemaline bodies and (iv) disruption of sarcomeric structures that are not observed in b) *neb*^*+/+*^ wildtype siblings

NEB nemaline myopathy is characterized by the presence of electron-dense nemaline bodies and myofibrillar disorganization, which have been observed in both patient muscle biopsies [21, 30] and mice models carrying mutations in *Neb* [3, 29]. To test whether these are present in *neb*^*−/−*^ zebrafish, we first stained sections of skeletal muscle at 6 dpf with Gomori trichome. We observed large darkly stained patches throughout the muscle fibers, indicative of nemaline bodies which were not present in stained sections from *neb*^*+/+*^ siblings (Fig. 2a). We also determined that *neb*^*−/−*^ fish have a much smaller skeletal muscle fiber cross-sectional area than *neb*^*+/+*^ siblings, reflecting a severe reduction in the diameter of muscle fibers (*neb*^*−/−*^ = 0.73±0.20 SD, *neb*^*+/+*^ = 1.0±0.29 SD) (Fig. 2b). We performed electron microscopy to analyze the muscle ultrastructure. As shown in Fig. 3a, we observed thickened Z-disks (Fig. 3ai) and an accumulation of electron dense nemaline bodies (Fig. 3aiii) in *neb*^*−/−*^ zebrafish at 6 dpf which were not present in wildtype siblings (Fig. 3b). *neb*^*−/−*^ mutant fibers also appeared disorganized and in many cases, there was a complete loss of structure with remnants of sarcomeric material (Fig. 3aii, aiv), suggesting a complete loss of muscle integrity.

We have previously shown that knockdown of Neb using two different antisense morpholinos produced Actinin2 and F-actin positive aggregates in the skeletal muscle [38]. We performed antibody staining for Actinin2 and F-actin and observed Actinin2 and F-actin positive aggregates along the vertical myosepta and scattered throughout *neb*^*−/−*^ skeletal muscle fibers at 2 dpf, similar to those identified in Neb morphants [38], which are not present in *neb*^*+/+*^ siblings (Fig. 2c). These actin accumulations do not result from broken fibers since the fibers in the same cell are intact (Fig. 2c inset). We also crossed our *neb* mutant strain to the Tg(*Lifeact-eGFP*) transgenic line, which labels actin in all of the thin filaments within the skeletal muscle. Unlike their wildtype siblings, Tg(*neb*^*−/−*^*; Lifeact-eGFP*) zebrafish show an accumulation of actin at the myosepta from 2 dpf and by 6 dpf, exhibit broken fibers greatly disrupting skeletal muscle structure (Additional file 1: Figure S1).

### Evaluation of nemaline myopathy treatments on *neb* mutants

We have established that the *neb*^*−/−*^ mutant zebrafish closely mimics nemaline myopathy phenotypes observed in patients validating its use as a NEB nemaline myopathy model. We next wanted to use the model to determine the efficacy of a number of suggested treatments which are currently self-administered by patients to improve skeletal muscle function. We selected four treatments (L-tyrosine, L-carnitine, creatine, and taurine) based on anecdotal reports from the nemaline myopathy patient support website (http://www.nemaline.org/resources/drugs.html) and published studies on nemaline myopathy patients and mice models [15, 33].

Given that none of these compounds have been previously tested in zebrafish, we first determined a maximal dose to investigate therapeutic potential without affecting the health and viability of the fish. We chose six doses ranging from 0.1 μM to 10 mM, dissolved in water and added these to zebrafish embryo medium. For control treatments, we added the equivalent volume of water, which was used as the vehicle, to the zebrafish embryo medium instead of the compound. For each of the treatments, we recorded the survival from 24 hpf to 6 dpf as well as the resting heart rate of the embryos at 2 dpf. We also quantified the swimming performance at 6 dpf and, combined with the survival and heart rate results, determined that a L-tyrosine and L-carnitine concentration of 10 μM (for L-tyrosine: [23], for L-carnitine: Additional file 1: Figure S3), a taurine concentration of 1 mM (Additional file 1: Figure S2), and a creatine concentration of 100 μM (Additional file 1: Figure S4) are the maximal non-toxic doses for treatment.

**Fig. 4:**
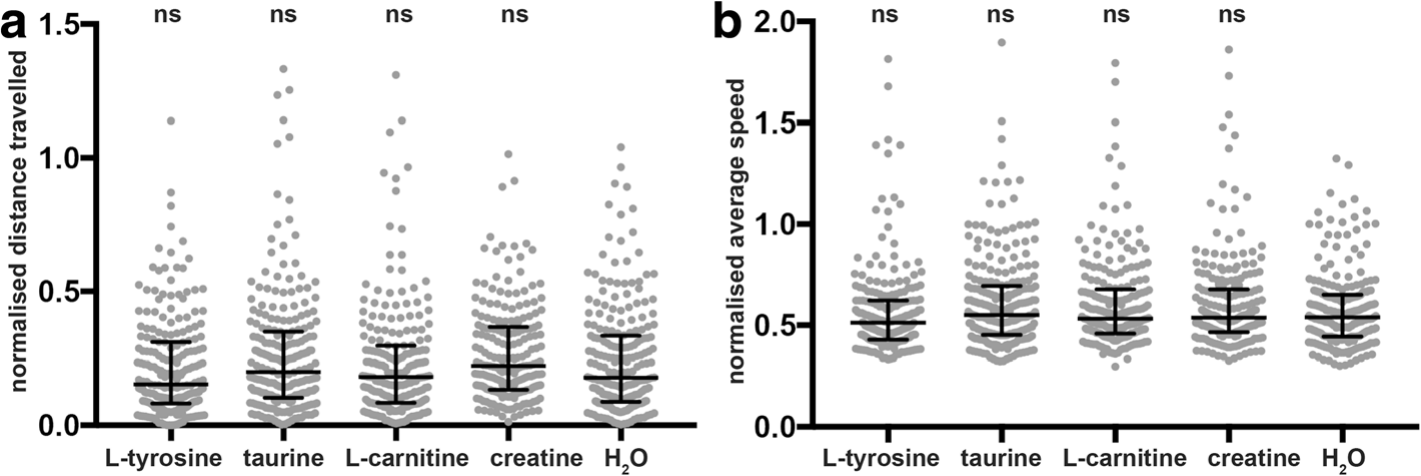
Quantification of muscle function in *neb*^*−/−*^ mutants at 6 dpf. Quantification of the **a**) normalized distance and **b**) speed travelled by *neb*^*−/−*^ mutants at 6 dpf supplemented with either L-tyrosine, taurine, L-carnitine, creatine, or water (H_2_O). Error bars represent median±interquartile range for three independent experiments (for a; *n* = 81,79,51 *neb*^*−/−*^ for L-tyrosine; *n* = 82,89,59 *neb*^*−/−*^ for taurine; n = 82,82,42 *neb*^*−/−*^ for L-carnitine; *n* = 87,79,46 *neb*^*−/−*^ for creatine, and n = 87,96,42 *neb*^*−/−*^ for water and for b; n = 81,79,51 *neb*^*−/−*^ for L-tyrosine; n = 82,89,59 *neb*^*−/−*^ for taurine; n = 82,82,42 *neb*^*−/−*^ for L-carnitine; *n* = 92,79,46 *neb*^*−/−*^ for creatine, and n = 87,96,42 *neb*^*−/−*^ for water per experiment). ns = not significant

To determine whether any of the treatments improved skeletal muscle function or pathology we quantified both the locomotion and phenotypic severity of the skeletal muscle in the treated NEB nemaline model fish at 6 dpf. We observed a significant decrease in swimming performance for *neb*^*−/−*^ mutants compared to their wildtype siblings (*neb*^*+/+*^ and *neb*^*+/−*^) (Additional file 1: Figure S5 and S6). However, for all chemicals tested there was no significant difference in the distance or speed travelled by *neb*^*−/−*^ mutants treated with the chemical supplements compared to water treated *neb*^*−/−*^ fish (Fig. 4).

**Fig. 5:**
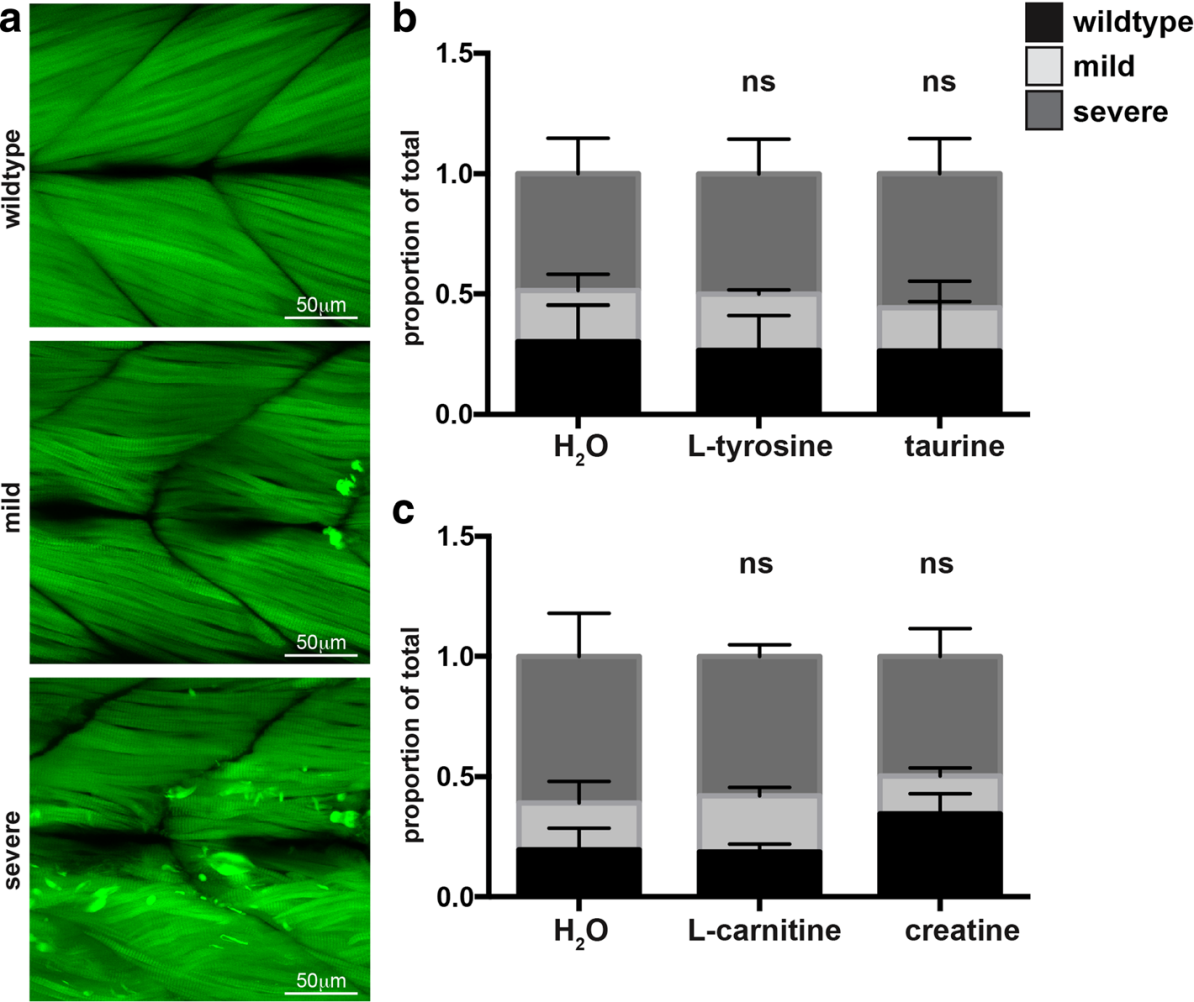
Quantification of the phenotypic severity of *neb*^*−/−*^ mutants at 6 dpf. Quantification of the phenotypic severity of Tg(*neb*^*−/−*^*; Lifeact-eGFP*) fish at 6 dpf supplemented with either L-tyrosine, taurine, L-carnitine, creatine, or water (H_2_O). **a** Phenotypes were scored as either wildtype, mild (less than five Lifeact-eGFP positive aggregates at the myosepta or a mild disruption of muscle fibres), or severe (severely disorganised fibres or an accumulation of five or more Lifeact-eGFP positive aggregates within the muscle cell). **b** Quantification of the phenotypic severity of Tg(*neb*^*−/−*^*; Lifeact-eGFP*) fish supplemented with either L-tyrosine, taurine, or water. **c** Quantification of the phenotypic severity of Tg(*neb*^*−/−*^*; Lifeact-eGFP*) fish supplemented with either L-carnitine, creatine, or water (H_2_O). **b** & **c** Error bars represent mean±SEM for three independent experiments. For **b**) *n* = 6,8,7 Tg(*neb*^*−/−*^*; Lifeact-eGFP*) for L-tyrosine, n = 11,5,11 Tg(*neb*^*−/−*^*; Lifeact-eGFP*) for taurine and n = 9,8,10 Tg(*neb*^*−/−*^*; Lifeact-eGFP*) for water. For **c**) n = 8,10,4 Tg(*neb*^*−/−*^*; Lifeact-eGFP*) for L-carnitine, n = 6,8,3 Tg(*neb*^*−/−*^*; Lifeact-eGFP*) for creatine, and *n* = 10,9,5 Tg(*neb*^*−/−*^*; Lifeact-eGFP*) for water per experiment). ns = not significant

To assess pathology in treated *neb*^*−/−*^ zebrafish as well as their wildtype siblings, we categorized the phenotypic severity of the skeletal muscle as wildtype, mild (less than five aggregates at the myosepta) and severe (fiber breakage and more than five aggregates at the myosepta and throughout the muscle fibers; Fig. 5a) and then genotyped the scored the embryos. We found no significant difference in the severity of the skeletal muscle phenotype of *neb*^*−/−*^ mutants, nor for their wildtype siblings for all treatments tested (Fig. 5b+c), showing these therapies do not improve skeletal muscle function in our nemaline myopathy model. There was also no obvious difference in the appearance of facial muscles between *neb*^*+/+*^ siblings and *neb*^*−/−*^ mutants. (Additional file 1: Figure S8).

## Discussion

At present, there is no effective therapy for nemaline myopathies with limited research into treatments in vivo using animal systems. For individuals surviving through childhood, nemaline myopathy is a chronic condition requiring continued therapy throughout life to manage symptoms. Our main goal is to develop zebrafish nemaline myopathy models to find effective treatments that can improve skeletal muscle function.

Here, we have characterized and validated a new NEB nemaline myopathy model containing a mutation within the super repeat region of the nebulin protein [9], causing nonsense mediating decay of the resulting transcript. Unlike the previously published model containing a missense mutation in the super repeat region, also predicted to cause a loss of nebulin protein function, we show that *neb*^*−/−*^ zebrafish mutants display darkly stained patches of muscle in Gomori trichome stained sections corresponding to nemaline bodies. Electron microscopy revealed the presence of electron dense nemaline bodies and thickened Z-disks consistent with those observed in patient biopsies [10, 21] which are not present in the previously published zebrafish *neb* mutant [39]. These pathological defects lead to a disruption in fiber integrity, indicated by the absence of sarcomeric structures by EM and loss of birefringence, resulting in reduced skeletal muscle function.

Akin to Neb KO mice models [3], *neb*^*−/−*^ mutant zebrafish are indistinguishable from their wildtype siblings prior to 2 dpf, however, as development proceeds their growth is reduced. The reduced growth in Neb KO and Neb^ΔExon55^ mice [20, 29] results from a significant reduction in thin filament length and fiber cross sectional area leading to a reduction in maximal force generating capacity [3, 20, 29] causing muscle weakness [30]. Similarly, sectioning of *neb*^*−/−*^ zebrafish larvae at 6 dpf revealed significantly smaller fiber cross-sectional area compared to wildtype siblings, indicating a reduction in fiber size. Both our Neb morphant model [38] and *neb*^*−/−*^ genetic mutants show an accumulation of actin at the myosepta suggesting that this may be an important pathological hallmark of nemaline myopathy. Thus, it is likely that the depletion of actin from the sarcomere, in addition to the loss of nebulin from the thin filament, may contribute to the reduction in myofiber size and the observed muscle weakness.

We evaluated a number of existing treatments for nemaline myopathy by quantifying their ability to reduce actin aggregation or improve skeletal muscle performance in our *neb*^*−/−*^ zebrafish. Of the four treatments we examined, L-tyrosine has been tested in ACTA1^H40Y^ mouse models [26] and in a limited patient study [15, 33] however no firm conclusions had been made as to its effectiveness to increase skeletal muscle function. Our results suggest that L-tyrosine treatment of *neb*^*−/−*^ mutant zebrafish, as reported for ACTA1^D286G^ zebrafish and mouse models [23], does not improve skeletal muscle performance. It is noted that L-tyrosine treatment may be beneficial to increase weight gain, appetite, and reduce pharyngeal secretions in nemaline myopathy patients [15, 33], however, this was not examined in the current study. We did however, examine the facial muscle structure in *neb*^*−/−*^ zebrafish, and observed no difference in appearance compared to their wild type siblings preventing an assessment of the effect of treatments on facial musculature.

We also treated *neb*^*−/−*^ mutants with either taurine, creatine, or L-carnitine, which are all naturally occurring compounds, present in many tissues including skeletal muscle, and are involved in modulating ion channel function, membrane stability, calcium homeostasis and energy metabolism [6, 7, 12, 13, 17, 22, 25, 42]. Taurine has been previously shown to improve muscle strength and reduce inflammation in mdx mice [40, 41] and in an analysis of six clinical trials for muscular dystrophy, 192 participants reported an increase in muscle strength when treated with creatine compared to the placebo group [16]. Unfortunately, chemical supplementation of *neb*^*−/−*^ mutants with creatine, L-carnitine, or taurine also failed to restore skeletal muscle function or improve skeletal muscle pathology. Interestingly, of the four compounds tested, taurine showed the least toxicity at high concentrations, which is in line with previous reports from clinical trials [36]. However, toxic effects observed in wildtype zebrafish treated with high doses of creatine, and L-carnitine suggest caution when administering high doses to patients. Importantly, our study has highlighted the inadequacies of existing nemaline myopathy treatments. Nevertheless, the zebrafish NEB nemaline myopathy model we have characterized provides an excellent system in which to perform high-throughput chemical screens and find an effective treatment for nemaline myopathy.

## Additional file

Additional file 1: Supplementary data for Testing of therapies in a novel nebulin nemaline myopathy model demonstrates and lack of efficacy. **Figure S1:** Characterisation of Tg(neb-/-; Lifeact-eGFP) fish. **Figure S2-S4:** Toxicity analyses for treatment of wildtype zebrafish with taurine, L-carnitine and creatine. **Figure S5-S6:** Quantification of distance travelled and average speed at 6 dpf. **Figure S7:** Quantification of the phenotypic severity at 6 dpf. **Figure S8:** Characterisation of facial muscles at 6 dpf. (PDF 7847 kb)
