# Supplementary material for: Testing of therapies in a novel nebulin nemaline myopathy model demonstrate a lack of efficacy
Source: Acta Neuropathol Commun. 2018 May 30;6:40. doi: 10.1186/s40478-018-0546-9 (PMC5977763; doi:10.1186/s40478-018-0546-9)
Supplement: Supplementary file 1 — Supplementary data for Testing of therapies in a novel nebulin nemaline myopathy model demonstrates and lack of efficacy. Figure S1: Characterisation of Tg(neb-/-; Lifeact-eGFP) fish. Figure S2-S4: Toxicity analyses for treatment of wildtype zebrafish with taurine, L-carnitine and creatine. Figure S5-S6: Quantification of distance travelled and average speed at 6 dpf. Figure S7: Quantification of the phenotypic severity at 6 dpf. Figure S8: Characterisation of facial muscles at 6 dpf. (PDF 7847 kb) [file 40478_2018_546_MOESM1_ESM.pdf]

<u>**Supplementary Information**</u>

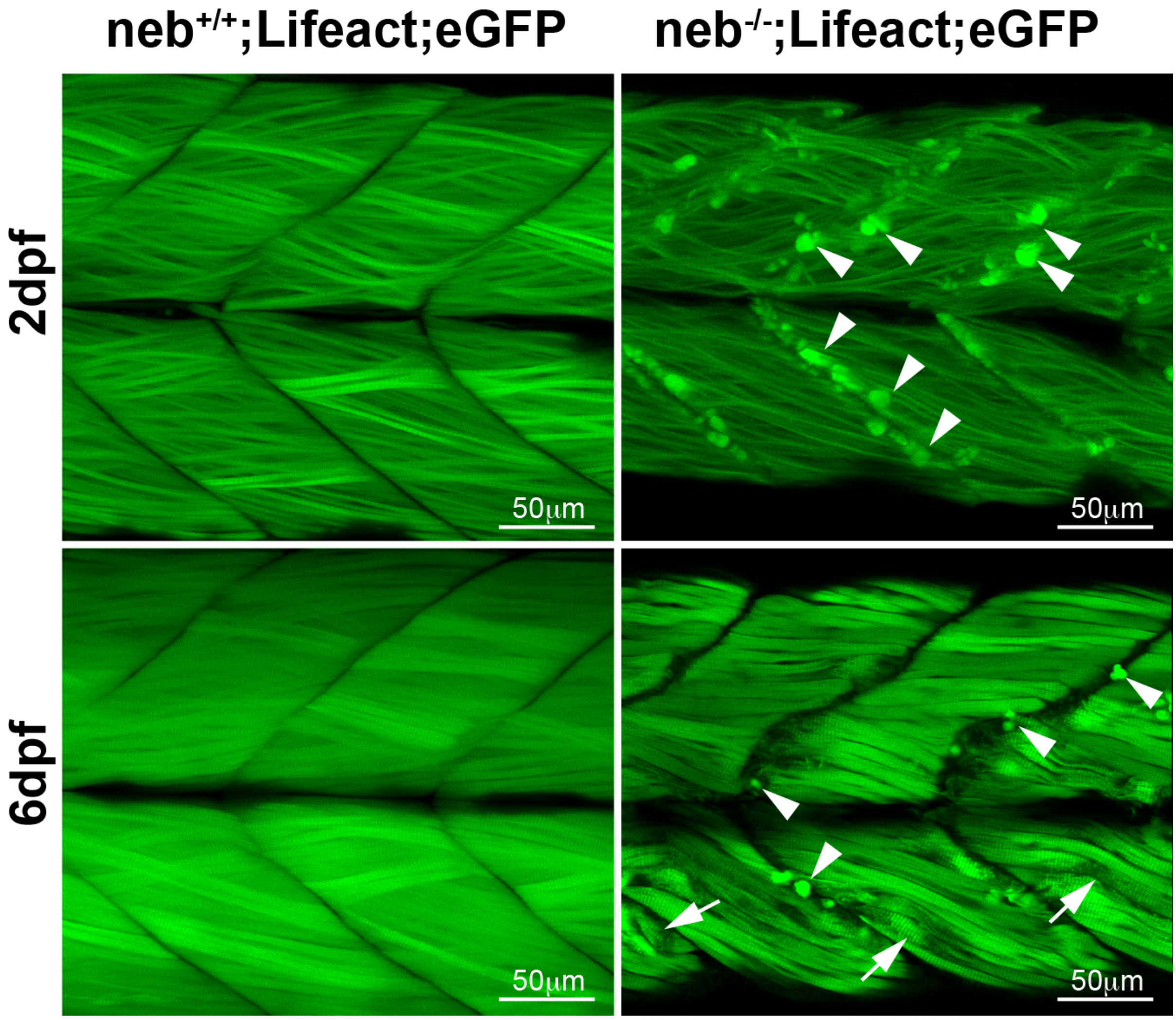

Fig S1: Characterisation of Tg(*neb*$^{-/-}$*; Lifeact-eGFP*) fish. Tg(*neb*$^{-/-}$*; Lifeact-eGFP*) fish show an accumulation of Lifeact-eGFP at the myosepta (arrowheads) at both 2 dpf and 6 dpf as well as regions of disorxganized muscle fibers (arrows) at 6 dpf that are not observed in Tg(*neb*$^{+/+}$*; Lifeact-eGFP*) siblings.

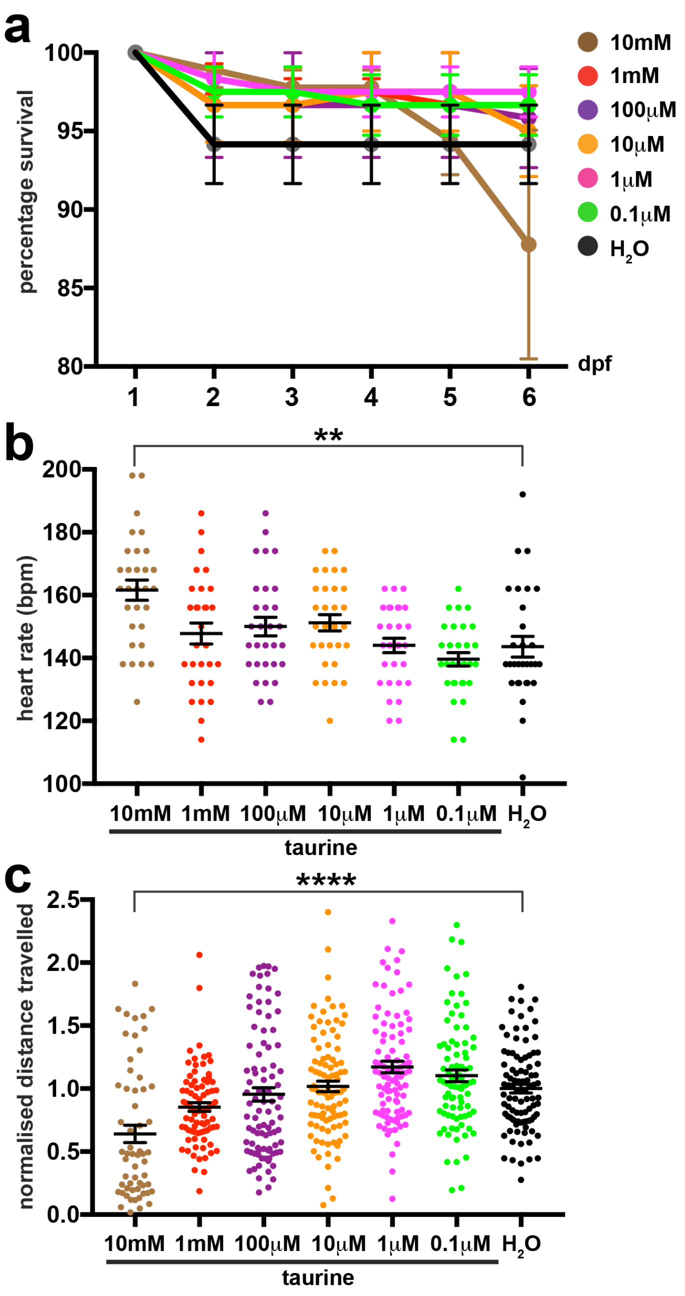

Fig S2: **Toxicity analyses for treatment of wildtype zebrafish with taurine**. a) Percentage survival and b) resting heart rate in beats per minute (bpm) of zebrafish treated with increasing taurine concentrations (from 0.1 μM to 10 mM) or water (H_2O). Error bars represent mean±SEM for three independent experiments with 30 zebrafish per experiment for survival assays and 10 zebrafish per experiment for heart rate assays, **$p<0.01$. c) Normalised distance travelled by 6 dpf zebrafish treated with increasing taurine concentrations (from 0.1 μM to 1 mM) or H_2O. Error bars represent mean±SEM for three independent experiments with n=15,22,21 for 10mM, n=23,24,24 for 1mM, n=19,24,24 for 100 μM, n=24,24,24 for 10 μM, n=24,23,24 for 1 μM, n=24,24,24 for 0.1 μM and n=24,23,23 for H_2O per experiment, ****$p<0.0001$.

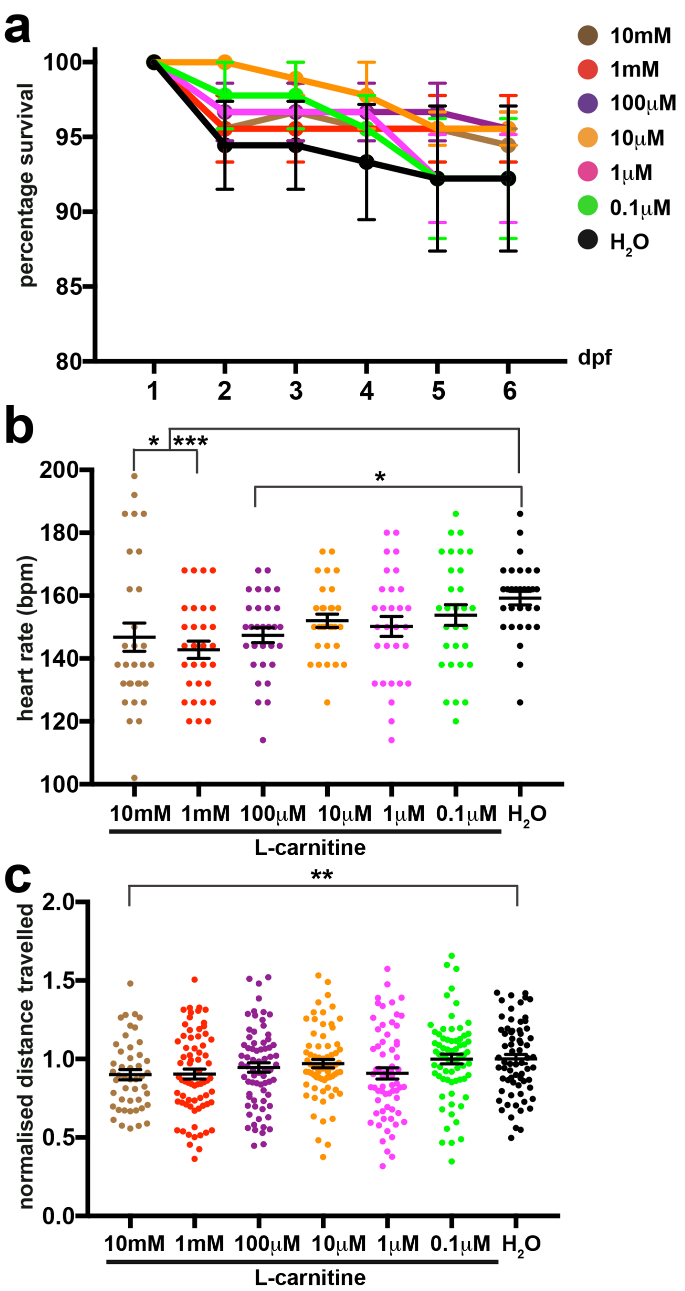

Fig S3: **Toxicity analyses for treatment of wildtype zebrafish with L-carnitine**. a) Percentage survival and b) resting heart rate in beats per minute (bpm) of zebrafish treated with increasing L-carnitine concentrations (from 0.1 µM to 10 mM) or water (H_2O). Error bars represent mean±SEM for three independent experiments with 30 zebrafish per experiment for survival assays and 10 zebrafish per experiment for heart rate assays, *$p<0.05$ and ***$p<0.001$. c) Normalised distance travelled by 6 dpf zebrafish treated with increasing L-carnitine concentrations (from 0.1 µM to 1 mM) or water (H_2O). Error bars represent mean±SEM for three independent experiments with n=18,23,6 for 10mM, n=24,23,23 for 1mM, n=24,24,23 for 100 µM, n=24,24,23 for 10 µM, n=23,16,23 for 1 µM, n=24,23,23 for 0.1 µM and n=22,24,22 for H_2O per experiment, **$p<0.01$.

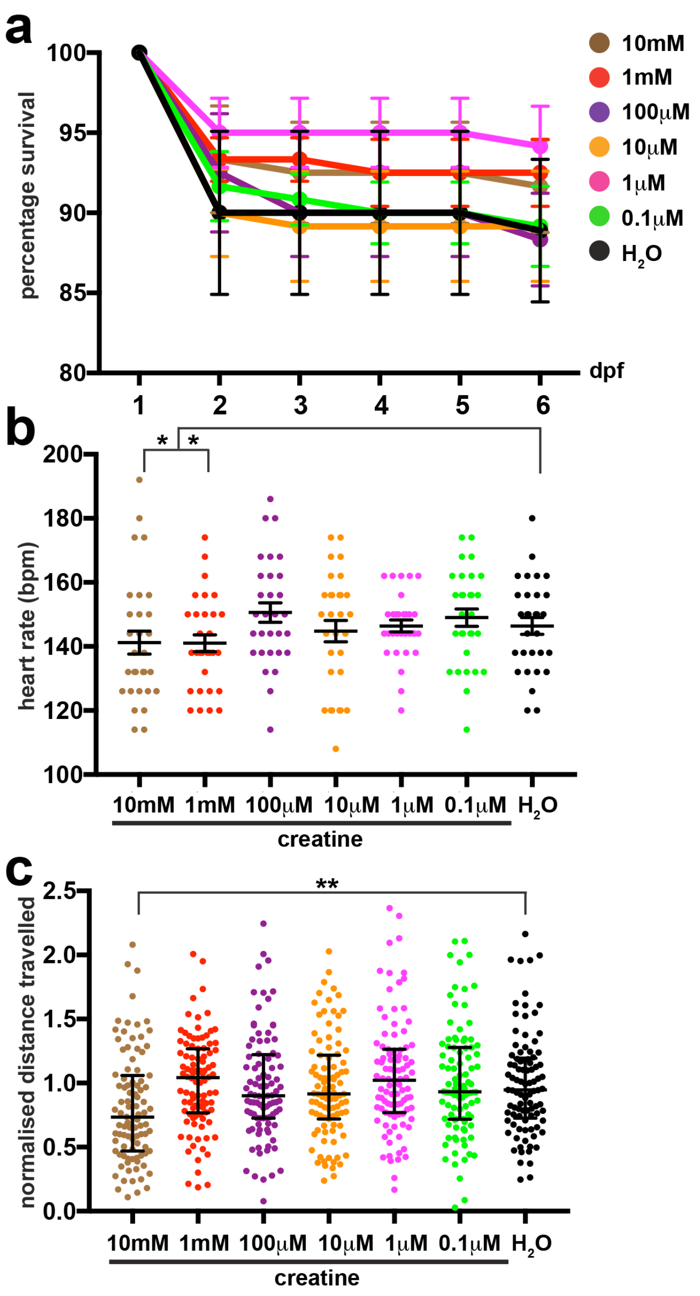

Fig S4: **Toxicity analyses for treatment of wildtype zebrafish with creatine**. a) Percentage survival and b) resting heart rate in beats per minute (bpm) of zebrafish treated with increasing creatine concentrations (from 0.1 µM to 10 mM) or water (H_2O). Error bars represent mean±SEM for three independent experiments with 30 zebrafish per experiment for survival assays and 10 zebrafish per experiment for heart rate assays, *$p<0.05$. c) Normalised distance travelled by 6 dpf zebrafish treated with increasing creatine concentrations (from 0.1 µM to 1 mM) or water (H_2O). Error bars represent median±interquartile range for four independent experiments with n=24,23,23,24 for 10mM, n=24,22,24,24 for 1mM, n=24,24,22,23 for 100 µM, n=24,24,24,23 for 10 µM, n=24,24,24,23 for 1 µM, n=24,24,24,24 for 0.1 µM and n=24,23,23,23 for H_2O per experiment, **$p<0.01$.

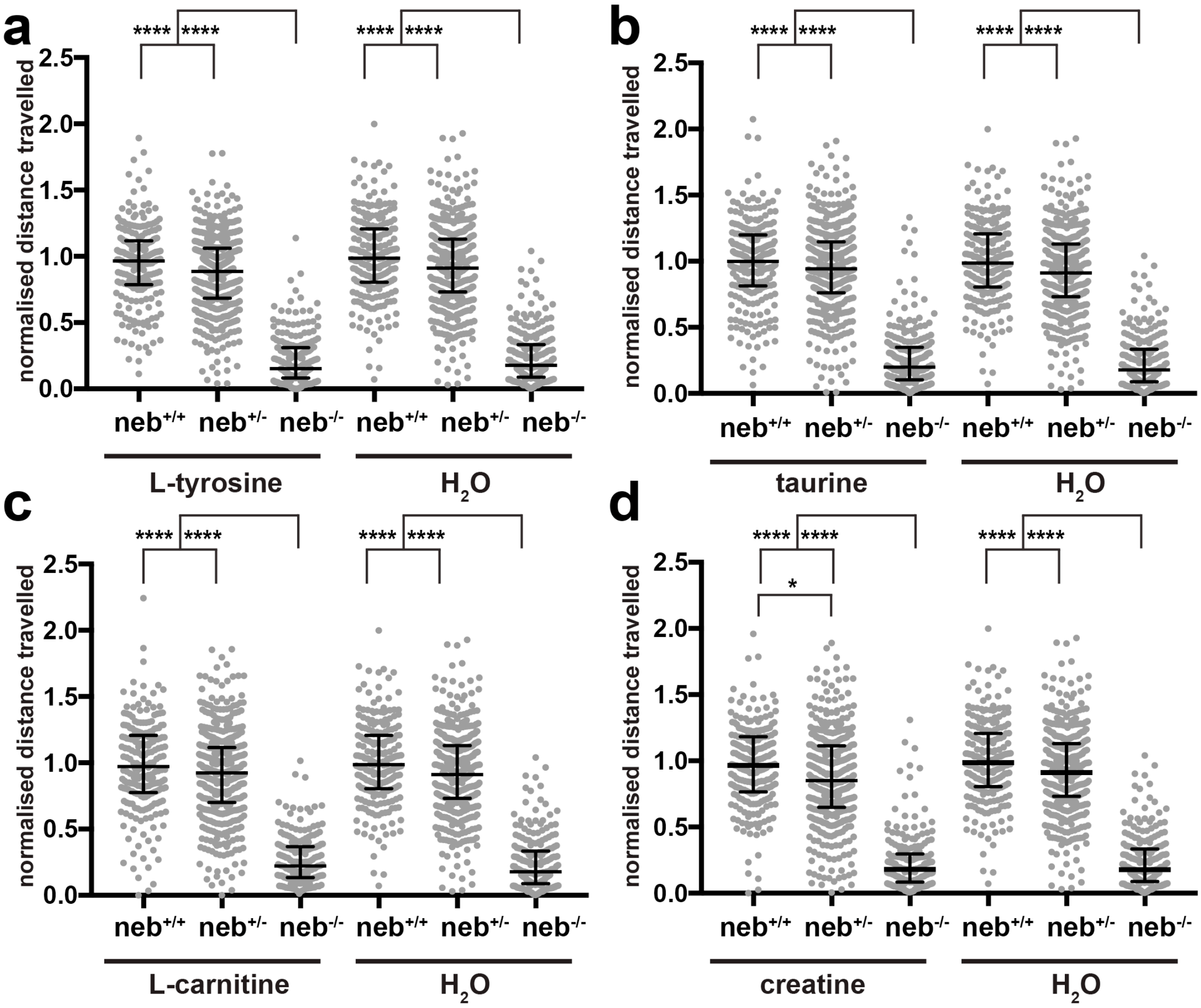

Fig S5: **Quantification of distance travelled by *neb*$^{-/-}$ mutants and wildtype siblings at 6 dpf**. Quantification of the normalized distance travelled by *neb*$^{-/-}$ mutants compared to wildtype siblings at 6 dpf supplemented with either a) L-tyrosine, b) taurine, c) L-carnitine, d) creatine, or water (H_2O). Error bars represent median±interquartile range for three independent experiments (n = 81,79,51 *neb*$^{-/-}$, 187,185,107 *neb*$^{+/-}$, 85,82,52 *neb*$^{+/+}$ for L-tyrosine; n = 82,89,59 *neb*$^{-/-}$, 183,158,94 *neb*$^{+/-}$, 95,101,42 *neb*$^{+/+}$ for taurine; n = 90,86,40 *neb*$^{-/-}$, 179,172,107 *neb*$^{+/-}$, 87,79,46 *neb*$^{+/+}$ for L-carnitine; n = 82,82,42 *neb*$^{-/-}$, 189,174,97 *neb*$^{+/-}$, 86,86,52 *neb*$^{+/+}$ for creatine, and n = 87,96,42 *neb*$^{-/-}$, 171,165,102 *neb*$^{+/-}$, 89,94,51 *neb*$^{+/+}$ for water per experiment), ****$p<0.0001$.

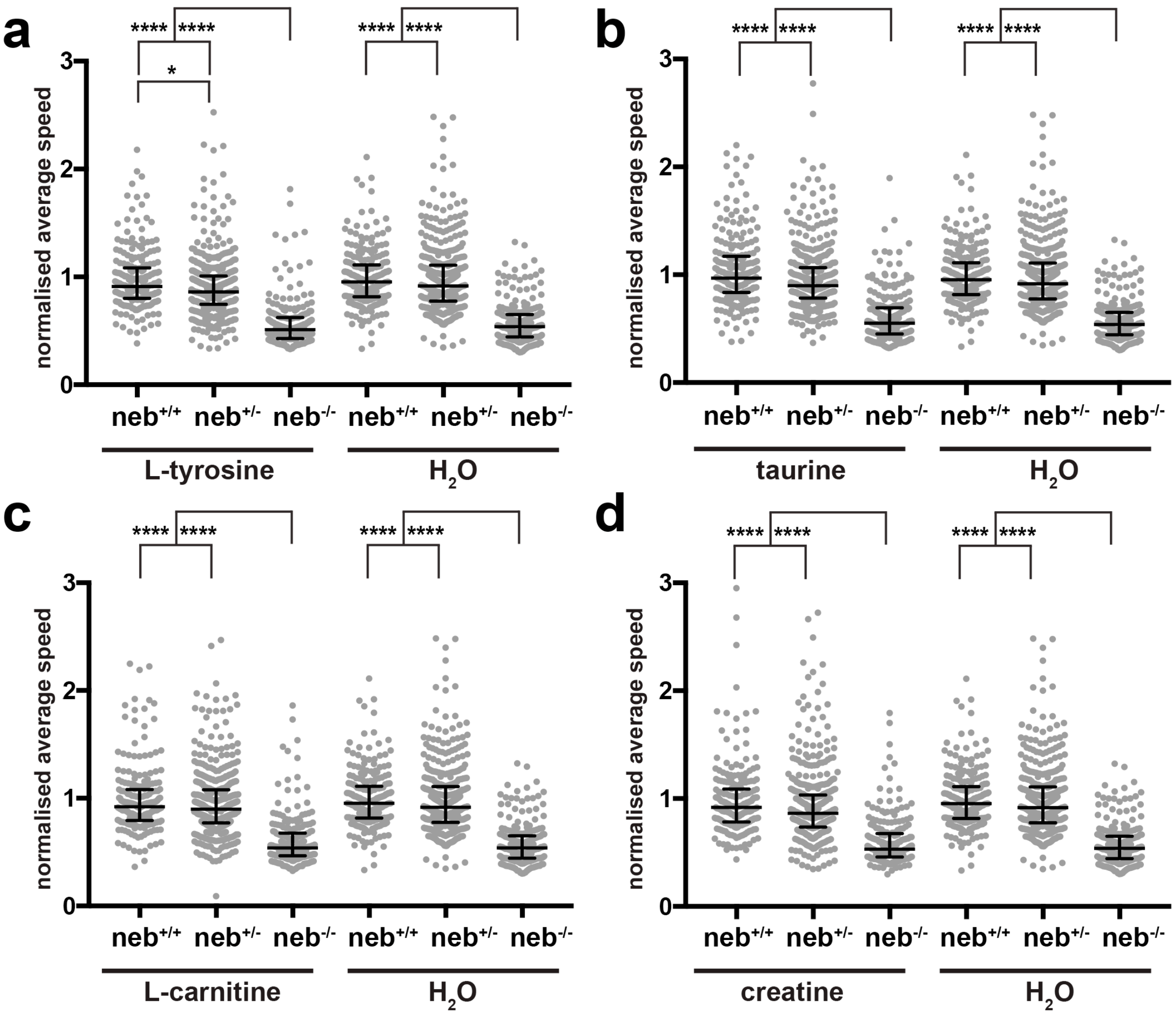

Fig S6: **Quantification of speed travelled by *neb*$^{-/-}$ mutants and wildtype siblings at 6 dpf**. Quantification of the normalized speed travelled by *neb*$^{-/-}$ mutants compared to wildtype siblings at 6 dpf supplemented with either a) L-tyrosine, b) taurine, c) L-carnitine, d) creatine, or water (H_2O). *Error bars* represent median±interquartile range for three independent experiments (n = 81,79,51 *neb*$^{-/-}$, 187,185,107 *neb*$^{+/-}$, 85,82,52 *neb*$^{+/+}$ for L-tyrosine; n = 82,89,59 *neb*$^{-/-}$, 183,158,94 *neb*$^{+/-}$, 95,101,42 *neb*$^{+/+}$ for taurine; n = 92,79,46 *neb*$^{-/-}$, 179,172,107 *neb*$^{+/-}$, 90,86,40 *neb*$^{+/+}$ for L-carnitine; n = 82,82,42 *neb*$^{-/-}$, 189,174,97 *neb*$^{+/-}$, 86,86,52 *neb*$^{+/+}$ for creatine and n = 87,96,42 *neb*$^{-/-}$, 171,165,102 *neb*$^{+/-}$, 89,94,51 *neb*$^{+/+}$ for water per experiment), *$p<0.05$ and ****$p<0.0001$.

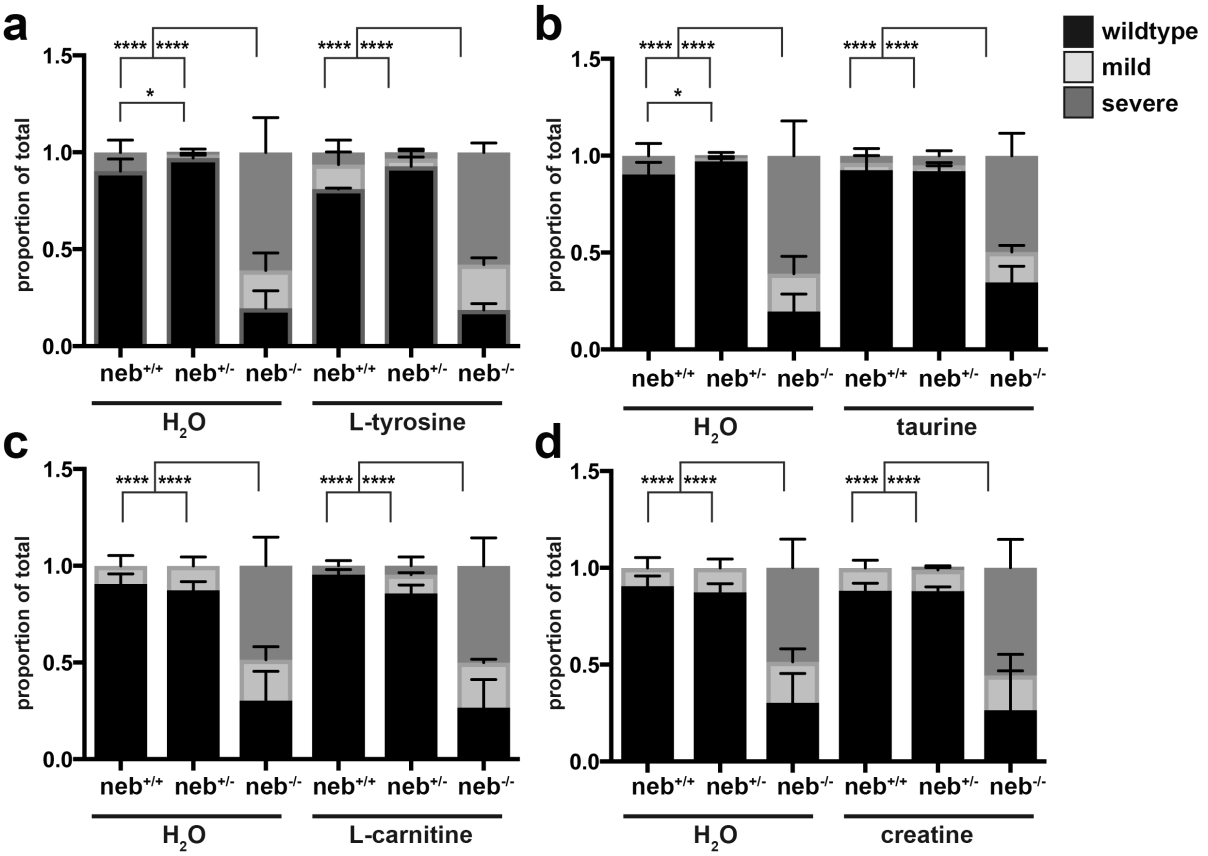

Fig S7: **Quantification of the phenotypic severity of *neb*$^{-/-}$ mutants and wildtype siblings at 6 dpf**. Quantification of the phenotypic severity of Tg(*neb*$^{-/-}$*; Lifeact-eGFP*) fish and their wildtype siblings supplemented with either a) L-tyrosine, b) taurine, c) L-carnitine, d) creatine or water (H_2O). Error bars represent mean±SEM for three independent experiments (For a & b) n = 6,8,7 Tg(*neb*$^{-/-}$*; Lifeact-eGFP*), 18,18,25 Tg(*neb*$^{+/-}$*; Lifeact-eGFP*), 16,10,11 Tg(*neb*$^{+/+}$*; Lifeact-eGFP*) for L-tyrosine, n = 11,5,11 Tg(*neb*$^{-/-}$*; Lifeact-eGFP*), 16,23,23 Tg(*neb*$^{+/-}$*; Lifeact-eGFP*), 7,9,11 Tg(*neb*$^{+/+}$*; Lifeact-eGFP*) for taurine; and n = 9,8,10 Tg(*neb*$^{-/-}$*; Lifeact-eGFP*), 27,20,14 Tg(*neb*$^{+/-}$*; Lifeact-eGFP*), 14,11,13 Tg(*neb*$^{+/+}$*; Lifeact-eGFP*) for water. For C & D) n = 8,10,4 Tg(*neb*$^{-/-}$*; Lifeact-eGFP*), 24,22,17 Tg(*neb*$^{+/-}$*; Lifeact-eGFP*), 11,6,21 Tg(*neb*$^{+/+}$*; Lifeact-eGFP*) for L-carnitine, n = 6,8,3 Tg(*neb*$^{-/-}$*; Lifeact-eGFP*), 30,22,19 Tg(*neb*$^{+/-}$*; Lifeact-eGFP*), 8,11,21 Tg(*neb*$^{+/+}$*; Lifeact-eGFP*) for creatine; and n = 10,9,5 Tg(*neb*$^{-/-}$*; Lifeact-eGFP*), 16,20,29 Tg(*neb*$^{+/-}$*; Lifeact-eGFP*), 15,10,11 Tg(*neb*$^{+/+}$*; Lifeact-eGFP*) for water per experiment), *$p<0.05$ and ****$p<0.0001$.

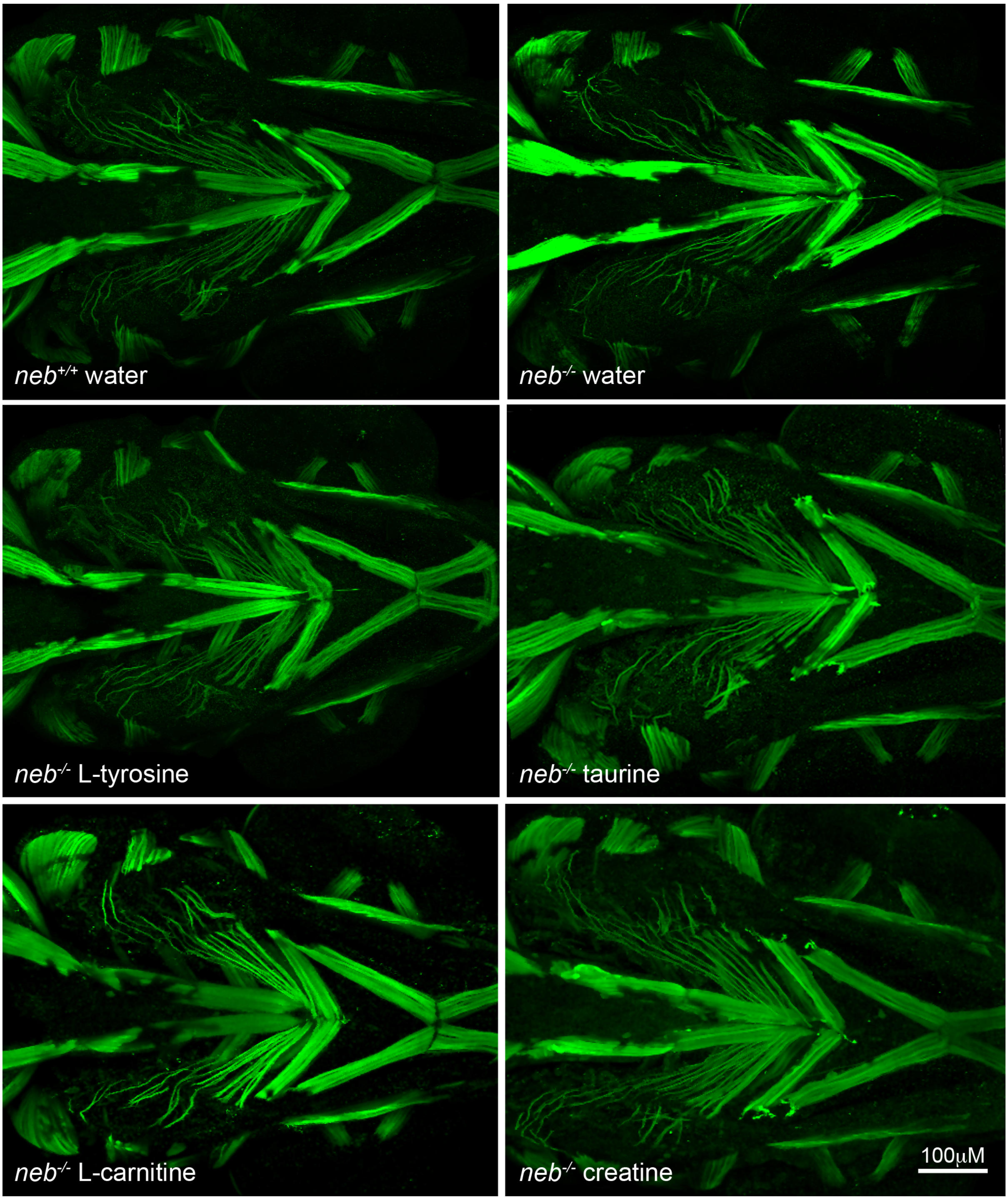

Fig S8: **Characterisation of facial muscles in Tg(*neb*$^{-/-}$; *Lifeact-eGFP*) fish and wild type siblings at 6 dpf.** Maximum projection images of Tg(*neb*$^{-/-}$; *Lifeact-eGFP*) fish supplemented with water, tyrosine, taurine, L-carnitine of creatine show no difference in the appearance of facial muscles to Tg(*neb*$^{+/+}$; *Lifeact-eGFP*) siblings supplemented with water.
